# Predictors of vitamin D deficiency in inflammatory bowel disease and health: A Mississippi perspective

**DOI:** 10.3748/wjg.v23.i4.638

**Published:** 2017-01-28

**Authors:** Kumar Pallav, Daniel Riche, Warren L May, Patrick Sanchez, Nitin K Gupta

**Affiliations:** Kumar Pallav, Patrick Sanchez, Nitin K Gupta, Division of Digestive Diseases, University of Mississippi Medical Center, Jackson, MS 39216, United States; Daniel Riche, Warren L May, Department of Pharmacy Practice, University of Mississippi Medical Center, Jackson, MS 39216, United States

**Keywords:** Vitamin D deficiency, Inflammatory bowel disease, Body mass index, Ulcerative colitis, Crohn’s disease, African American

## Abstract

**AIM:**

To identify the predictors of vitamin D deficiency in patients with and without inflammatory bowel disease (IBD).

**METHODS:**

Patients with ulcerative colitis (UC) or Crohn’s disease (CD) related diagnostic codes who received medical care at University of Mississippi Medical Center between July 2012 and 2015 were identified. After thorough chart review, we identified patients with biopsy proven IBD who had also been tested for serum 25-hydroxyvitamin D [25(OH)D] concentration. We compared these patients to a previously studied cohort of healthy controls who also had vitamin D concentration checked. Logistic regression analysis was performed to determine the association between vitamin d deficiency and UC, CD, race, age, gender and body mass index (BMI).

**RESULTS:**

We identified 237 patients with confirmed IBD. Of these, only 211 had a serum 25(OH)D concentrations available in the medical record. The group of healthy controls consisted of 98 individuals with available serum 25(OH)D concentration. 43% of IBD patients were African American (AA). Patients with CD were more likely to have vitamin D concentration checked. Bivariate analysis showed that AA (51% *vs* 21%, *P* = 0.00001), subjects with BMI >30 kg/m^2^ (39% *vs* 23% *P* = 0.01) and CD (40% *vs* 26%, *P* = 0.04) were more likely to be vitamin D deficient than vitamin D sufficient. Those with Age > 65 were more likely to be vitamin D sufficient (46% *vs* 15%, *P* = 0.04). Multiple regression showed that only BMI > 30 kg/m^2^ and AA race are associated with vitamin D deficiency.

**CONCLUSION:**

BMI > 30 kg/m^2^ and AA race are predictive of vitamin D deficiency. Gender, age and diagnosis of IBD are not predictive of vitamin D deficiency.

**Core tip:** The studies evaluating the relationship between vitamin D deficiency and inflammatory bowel disease (IBD) have shown heterogeneity perhaps due to multiple overlapping risk factors that need to be accounted for. We performed a retrospective study to identify the risk factors for vitamin D deficiency in a population with a large African American (AA) component. Using logistic regression analysis we studied the effect of diagnosis, race, age, gender and body mass index (BMI) on prevalence of vitamin D deficiency. In subjects with and without IBD, BMI > 30 kg/m^2^ and AA race are predictive of vitamin D deficiency. Gender, age and diagnosis of IBD were not predictive of vitamin D deficiency in our population.

## INTRODUCTION

Crohn’s disease (CD) and ulcerative colitis (UC) are chronic idiopathic conditions of the gastrointestinal tract that manifest as inflammation with distinct, yet often overlapping clinical features[1]. The Etiology of IBD is thought to reflect innate and adaptive immune-mediated responses to luminal bacterial antigens leading to enhanced intestinal permeability and dysregulated intestinal immunity[1-3]. There has been an increasing interest in the regulatory effects of vitamin D on the immune system in IBD and colorectal cancer[4,5]. Numerous studies have demonstrated a link between low serum 25-hydroxyvitamin D [25(OH)D] concentrations and IBD in both CD[6-10] and UC patients[10,11]. Vitamin D deficiency may predispose IBD patients to a higher disease activity[6], suboptimal response to treatment[12], and higher incidence of surgery and hospitalization[13]. On the other hand, as pointed out by Tajika et al[14], IBD patients may be at an increased risk for low serum 25(OH)D concentrations due to one or more of the following: Faulty conversion of vitamin D to active metabolic forms; failure to conserve an adequate functional pool of vitamin D; Insufficient dietary intake and inadequate sun exposure[15,16]; malabsorption of dietary and biliary vitamin D and its metabolites[17,18]; and loss of protein-bound 25hydroxy vitamin D due to a protein losing enteropathy[19].

Studies aimed at delineating this complex relationship are confounded by factors such as age, BMI, and race leading to inconsistent conclusions[20-25]. Furthermore, African Americans (AA) are understudied in most of the IBD literature, and data representing this population is scarce[26]. According to the 2011 United States Census Bureau, 40% of Mississippians are AA[27] thereby presenting a unique opportunity to study this population.

We aim to determine the vitamin D status in an understudied cohort consisting of IBD and non-IBD patients and Investigate the association between serum 25(OH)D concentrations and IBD diagnosis (UC and CD). In addition, this study aimed to investigate risk factors for vitamin D deficiency namely race, gender, age, and BMI; as well as to compare vitamin D status with that of healthy controls.

## MATERIALS AND METHODS

This retrospective study was conducted at University of Mississippi Medical Center (UMMC), which is a tertiary care center and the only academic medical institution in the state of Mississippi. Over half a million patient encounters are reported every year. While UMMC caters to both high and low acuity patients, being a referral center more patients tend to be sicker. There are no reports for who sees most of the IBD patients in the state but we see over 500 patients annually. We get patients through word of mouth and community referrals. Less than 15% of the patients are uninsured and the majority has either public or private insurance. All patient visits are in the main campus in Jackson. Patients were identified using various diagnosis codes for UC, CD and IBD. Electronic medical records for all patients with IBD associated diagnostic codes seen between July 2012 and July 2015 were reviewed. Demographic, biometric, and clinical information was collected through review of electronic medical records. A standard document was used to collect the information however a pilot study was not conducted. Diagnosis of IBD was based on endoscopic, clinical and histologic data. IBD Patients with available plasma 25(OH)D concentration were included in this study. We excluded patients with history of malignancy. The control group consisted of patients without IBD or any active systemic disease that presented to UMMC and had plasma 25(OH)D concentrations obtained during routine follow up.

### Vitamin D status assessment

All vitamin D concentrations were assessed using The ARCHITECT 25-OH vitamin D assay (Abbott diagnostics, Germany). There is no absolute consensus on Vitamin D deficiency and sufficiency. Vitamin D was operationalized into clinically meaningful categories for analysis. Plasma 25(OH) D concentrations < 20 ng/mL (50 nmol/L) indicate vitamin D deficiency. Plasma 25(OH) D concentrations between 21 and 29 ng/mL (52.5 and 72.5 nmol/L) represent vitamin D insufficiency while concentrations > 30 ng/mL (75 nmol/L) represent vitamin D sufficiency[22,28,29].

### Statistical analysis

A biomedical statistician performed statistical analysis. We used a generalized logistic regression model to estimate odds ratio (OR). The generalized logistic regression extends the traditional model and in this instance, our outcome of interest was ordinal and has three levels for vitamin D: Deficient, insufficient and sufficient.

Comparisons of the distributions for demographic characteristics were made with Pearson’s χ^2^ statistic. Higher than expected Pearson’s residual (*i.e*., |Z|> 2.0) was considered evidence of departure from independence. We considered *P <* 0.05 evidence of statistical significance.

Data were reported as frequencies and proportions for the marginal distributions of the categorical variables and proportions for the joint distributions of the cross-classification tables. The institutional review board at UMMC approved this study.

## RESULTS

Two hundred and thirty seven IBD patients (139 CD, 98 UC) and 98 controls were identified. Amongst the IBD patients, 211 had 25(OH)D concentration checked on 257 occasions. Those with CD were more likely to have a 25(OH)D concentration measured in our facility. Also those tested for vitamin D concentration tended to be slightly older. Otherwise there were no major differences between IBD patients with and without measured 25(OH)D concentration (Table 1).

**Table 1:** Comparison between inflammatory bowel disease patients with and without available vitamin D concentration *n* (%)

	**IBD patients without available vitamin D concentration** **(*n* = 26)**	**IBD patients with available vitamin D concentration** **(*n* = 211)**	***P* value**
CD	10 (38.5)	129 (61.1)	0.034
Age (yr), median (IQR)	32 (26)	41 (25)	0.030
Female	12 (46.2)	125 (59.2)	0.213
AA	11 (42.3)	91 (43.1)	0.391
BMI (kg/m^2^), median (IQR)	25.6 (9.9)	27 (8.9)	0.176
Patients on vitamin D supplementation	2 (8.33)	36 (17.06)	0.271

CD: Crohn’s disease; AA: African American; BMI: Body mass index.

Of 309 patients included in final analysis, 98 (31.7%) were controls, 129 (41.7%) were CD patients and 82 (26.5%) were UC patients. Compared to IBD patients, the controls had higher mean age and female preponderance. IBD patients were more likely to be AA and had lower mean body mass index (BMI) (Table 2).

**Table 2:** Comparison between inflammatory bowel disease and non- inflammatory bowel disease patients *n* (%)

	**Controls**	**IBD patients**	***P* value**
	**(*n* = 98)**	**(*n* = 211)**	
Patients with vitamin D deficiency	56 (57.1)	143 (61.6)	0.0694
Age at vitamin D testing (yr), median (IQR)	60.5 (14.5)	41 (25)	< 0.0001
Female	86 (87.8)	125 (59.2)	< 0.0001
AA	23 (23.9)	91 (43.1)	0.0009
BMI (kg/m^2^), median (IQR)	29.3 (7.5)	27 (8.9)	0.0438
Patients receiving vitamin D supplementation	Not available	36 (17.06)	Not Applicable

AA: African American; BMI: Body mass index.

Demographics of the study population as a whole are shown in Table 3. Overall, there was a 2:1 female-to-male ratio. Within the IBD cohort, 115 (54.5%) subjects were White, 91 (43.1%) were AA and 5 (2.3%) were of other races. BMI was categorized into normal, overweight, and obese, with similar proportion of individuals in each category.

**Table 3:** Distribution of vitamin D concentration across various diagnosis, demographics (age, race, gender) and body mass index (modifiable risk factor) *n* (%)

		**Vitamin D**			***P* value**
		**Deficient**	**Insufficient**	**Sufficient**	
Total	309	100	99	110	
		(32.4)	(32.0)	(35.6)	
Diagnosis					
Controls	98 (31.7)	27.6%	29.6%	42.8%	0.0407
CD	129 (41.7)	40.3%	33.3%	26.4%	
UC	82 (26.5)	25.6%	32.9%	41.5%	
Age (yr)					
< 35	72 (23.3)	38.9%	34.7%	26.4%	0.0415
35-49	73 (23.6)	34.2%	28.8%	37.0%	
50-64	99 (32.0)	37.4%	28.3%	34.3%	
> 65	65 (21.0)	15.4%	38.5%	46.2%	
Race					
White	189 (61.2)	21.7%	34.4%	43.9%	< 0.0001
AA	114 (36.9)	50.9%	28.1%	21.0%	
Other	6 (1.9)	16.7%	33.3%	50.0%	
Gender					
Female	211 (68.3)	33.7%	32.2%	34.1%	0.6857
Male	98 (31.7)	29.6%	31.6%	38.8%	
BMI (kg/m^2^)					
< 25	97 (31.4)	29.9%	25.8%	44.3%	0.0110
25-30	102 (33.0)	27.5%	31.4%	41.2%	
> 30	110 (35.6)	39.1%	38.2%	22.7%	

CD: Crohn’s disease; AA: African American; BMI: Body mass index; UC: Ulcerative colitis.

Vitamin D as the outcome is also presented in Table 3 and divided into clinically meaningful categories. The marginal distribution of vitamin D given in the first row of Table 3 indicates that the sample is approximately evenly distributed with about one-third in each category.

### Bivariate analysis

Table 3 gives the results of a chi-square contingency table analysis to determine the association of vitamin D with each of the demographic variables.

Disease status (CD *vs* UC *vs* Control) and plasma vitamin D concentrations were significantly associated (*P =* 0.04). The proportion of controls with sufficient vitamin D was higher as compared to the other two groups. For the CD group, there were many more with deficient vitamin D than expected and fewer with sufficient vitamin D than expected.

Age and vitamin D were significantly associated (*P =* 0.041). The Pearson’s residuals indicated that the youngest age group (less than 35), had a higher proportion with deficient vitamin D than expected and a lower proportion of sufficient vitamin D than expected. The opposite was true for the age greater than 65 group where the proportion of those with deficient vitamin D was lower than expected, while the proportion in the sufficient group was higher than expected.

Race and vitamin D were significantly associated (*P <* 0.0001). The proportion of AA with deficient vitamin D was much higher than expected and the proportion with sufficient vitamin D was much lower than expected. Whites and others showed the opposite trend with lower than expected proportions with deficient vitamin D and higher than expected proportions with sufficient vitamin D.

Gender was not significantly associated with vitamin D sufficiency (*P =* 0.6).

BMI and vitamin D were significantly associated (*P =* 0.0110). For BMI < 25 kg/m^2^, the proportion of sufficient vitamin D subjects was higher than expected. Subjects with BMI > 30 kg/m^2^ had a higher proportion with deficient vitamin D than expected and a lower proportion with sufficient vitamin D than expected.

For all demographic variables, the insufficient group did not appear to differ significantly from the marginal of approximately one-third. The differences were in the sufficient and deficient vitamin D concentrations for diagnosis, Age, race and BMI.

Of the four factors that appeared to be associated with plasma vitamin D concentrations, BMI is the only modifiable risk factor. Therefore, we investigated the potential for confounding factors for the relationship of BMI with vitamin D by statistically testing the associations between BMI and non-modifiable risk factors: age, race and gender (Table 4).

**Table 4:** Associations of body mass index with diagnosis and demographic variables

	***n* (%)**	**BMI < 25 kg/m^2^**	**BMI 25-30 kg/m^2^**	**BMI > 30 kg/m^2^**	***P* value**
Total	309	97	102	110	
		(31.4)	(33.0)	(35.6)	
Diagnosis					
Controls	98 (31.7)	20.4%	34.7%	44.9%	0.0048
CD	129 (41.7)	42.6%	29.5%	27.9%	
UC	82 (26.5)	26.8%	36.6%	36.6%	
Age (yr)					
< 35	72 (23.3)	52.8%	29.2%	18.1%	0.0007
35-49	73 (23.6)	26.0%	30.1%	43.8%	
50-64	99 (32.0)	23.2%	37.4%	39.4%	
> 65	65 (21.0)	26.2%	33.9%	40.0%	
Race					
White	189 (61.2)	28.6%	36.5%	34.9%	0.5253
AA	114 (36.9)	36.0%	27.2%	36.8%	
Other	6 (1.9)	33.3%	33.3%	33.3%	
Gender					
Female	211 (68.3)	27.5%	30.3%	42.2%	0.0017
Male	98 (31.7)	39.8%	38.8%	21.4%	

CD: Crohn’s disease; AA: African American; BMI: Body mass index; UC: Ulcerative colitis.

BMI was associated with diagnosis (*P =* 0.0048), age (*P =* 0.0007) and gender (*P* = 0.0017). BMI was not significantly associated with race (Table 4).

### Distribution of vitamin D across stratified levels of BMI and diagnosis

**BMI < 25 kg/m^2^:** Those < 35 years old are more likely to have vitamin D deficiency. Curiously, the 50-64 year age group is less likely to exhibit vitamin D deficiency compared to the other groups. The CD patients are more likely to have deficient vitamin D than the other groups. There is a significant association between diagnosis and vitamin D only in the BMI < 25 kg/m^2^ group (*P =* 0.0026) (Table 5).

**Table 5:** Distribution of vitamin D concentration across stratified levels of body mass index and diagnosis

	***n* (%)**	**Vitamin D deficient**	**Vitamin D insufficient**	**Vitamin D sufficient**	***P* value**
BMI < 25 (kg/m^2^)					
Total	97	29	25	43	
		(29.9)	(25.8)	(44.3)	
Diagnosis					
Controls	20 (20.6)	15.0%	15.0%	70.0%	0.0026
CD	55 (56.7)	43.6%	27.3%	29.1%	
UC	22 (22.7)	9.1%	31.8%	59.1%	
BMI = 25-30 (kg/m^2^)					
Total	102	28	32	42	
		(27.5)	(31.4)	(41.2)	
Diagnosis					
Controls	34 (33.3)	26.5%	26.5%	47.1%	0.3894
CD	38 (37.3)	34.2%	36.8%	29.0%	
UC	30 (29.4)	20.0%	30.0%	50.0%	
BMI > 30 (kg/m^2^)					
Total	110	43	42	25	
		(39.1)	(38.2)	(22.7)	
Diagnosis					
Controls	44 (40.0)	34.1%	38.6%	27.3%	0.8823
CD	36 (32.7)	41.7%	38.9%	19.4%	
UC	30 (27.3)	43.3%	36.7%	20.0%	

CD: Crohn’s disease; AA: African American; BMI: Body mass index; UC: Ulcerative colitis.

**BMI 25-30 kg/m^2^:** No association was found in the BMI 25-30 kg/m^2^ group (*P =* 0.389) (Table 5).

**BMI > 30 kg/m^2^:** The BMI >30 kg/m^2^ group is the most homogeneous, and there is no statistical evidence of an association (*P =* 0.88).

That is, the BMI > 30 kg/m^2^ group is more likely to be vitamin D deficient, but there is no further evidence of a relationship between diagnosis and vitamin D once BMI > 30 kg/m^2^ is considered. On the other hand, the BMI < 25 kg/m^2^ group is more likely to have sufficient vitamin D, but the presence of CD may alter the effect on vitamin D. We find this group stands out as being vitamin D deficient compared to the others (Table 5).

### Seasonal variation in vitamin D concentrations

We compared mean vitamin D concentrations in traditional summer *vs* winter months and while the summertime vitamin D concentrations were marginally higher (26.9 *vs* 23.1, *P =* 0.064), this is not statistically significant. Admittedly, it was close enough to consider adding into the regression analysis, however this p-value is only using a partial dataset (based on seasonal analysis many patients are excluded) - therefore not included into the regression analysis.

### Regression model

We also investigated a cumulative logistic regression model that included age and race as covariates and the nine BMI categories. We did not include gender since it was not significantly associated with outcome or predictor. The model found race remained significant (*P =* 0.0006), age was not significant (*P =* 0.15), and there were significant differences between the nine BMI groups. To adjust for the multiple testing, we considered the proportions different only if *P <* 0.002, a conservative Bonferroni approach. Differences identified were the same as the stratified analysis in Table 6.

**Table 6:** Results of multivariate modelling with age, race, gender, body mass index and diagnosis as predictors of deficient, insufficient and sufficient vitamin D

	**Full model**		**Global**	**Reduced model**		**Global**
	**OR**	**OR**	***P* value (full)**	**OR**	**OR**	***P* value (reduced)**
	**(95%CI),**	**(95%CI),**		**(95%CI),**	**(95%CI),**	
	**deficient *vs* sufficient**	**insufficient *vs* sufficient**		**deficient *vs* sufficient**	**insufficient *vs* sufficient**	
Diagnosis			0.1482			0.0852
Controls	Ref.	Ref.		Ref.	Ref.	
CD	1.71	2.11		2.22	2.16	
	(0.74, 3.94)	(0.95, 4.69)		(1.07, 4.63)	(1.07, 4.36)	
UC	0.73	1.13		0.92	1.2	
	(0.30, 1.76)	(0.50, 2.53)		(0.42, 2.02)	(0.59, 2.48)	
Gender			0.9584			
Female	Ref.	Ref.				
Male	0.9	0.95				
	(0.46, 1.79)	(0.50, 1.81)				
Age (yr)			0.1578			
< 35	3.62	1.47				
	(1.18, 11.12)	(0.56, 3.83)				
35-49	1.9	0.68				
	(0.68, 5.30)	(0.28, 1.63)				
50-64	2.61	0.86				
	(1.04, 6.58)	(0.40, 1.86)				
> 65	Ref.	Ref.				
Race			0.0006^1^			< 0.0001^1^
AA	Ref.	Ref.		Ref.	Ref.	
White	0.25^1^	0.67		0.23^1^	0.64	
	(0.13, 0.48)	(0.34, 1.29)		(0.12, 0.43)	(0.33, 1.22)	
Other	0.3	0.68		0.18	0.57	
	(0.03, 3.38)	(0.09, 4.89)		(0.02, 1.95)	(0.08, 3.96)	
BMI (kg/m^2^)			0.0017^1^			0.0030^1^
< 25	0.68	0.57		0.71	0.63	
	(0.32, 1.43)	(0.28, 1.17)		(0.34, 1.48)	(0.31, 1.26)	
25-30	Ref.	Ref.		Ref.	Ref.	
> 30	2.71^1^	2.36^1^		2.61^1^	2.27^1^	
	(1.28, 5.73)	(1.17, 4.75)		(1.26, 5.42)	(1.14, 4.52)	

1 Statistically significant. CD: Crohn’s disease; AA: African American; BMI: Body mass index; UC: Ulcerative colitis.

### Reduced model

We performed a manual backward elimination procedure to reduce the model. For the procedure, we retained variables with *P <* 0.10. The reduced model includes race, BMI and diagnosis only. Table 6 gives the results of the analyses, both full and reduced model for comparison. The results are similar for both models; therefore, we interpret the odds ratios from the final model, only.

Although there was no significant effect for diagnosis at the *P <* 0.05 level, we did find a global difference (*P =* 0.085) and retained it in the model. Using a simple confidence interval for the odds ratio, the odds of deficiency compared to sufficiency are higher for the CD group compared to controls [odds ratio (OR) = 2.22; 95%CI: 1.07-4.63]. There was also a similar result for insufficiency compared to sufficiency comparing CD to controls (OR = 2.16; 95%CI: 1.07-4.36).

Race was a significant predictor of vitamin D concentrations based on the global test (*P <* 0.0001). Whites are about one-fourth less likely than AA to exhibit deficiency compared to sufficiency (OR = 0.23; 95%CI: 0.12-0.43). No other results are significant and the sample size is very small for the “Other” group, leading to loss of power.

Finally, BMI was a significant predictor of vitamin D concentrations based on a significant global test (*P =* 0.003). Using the normal weight group as a reference (*i.e*., 25-30 kg/m^2^), we estimated odds ratios for underweight and overweight. Although there were no significant effects for underweight, there was a significant effect for the overweight (BMI > 30 kg/m^2^) group. The overweight group is much more likely to develop vitamin D deficiency (OR = 2.61; 95%CI 1.26-5.42), as well as insufficiency compared to sufficiency (OR = 2.27; 95%CI: 1.14-4.52).

## DISCUSSION

Ergocalciferol (vitamin D2), the predominant circulating and storage form of vitamin D[30] and cholecalciferol (vitamin D3) are obtained from diet or supplementation. Vitamin D3 is also formed in the skin *via* ultraviolet B (UVB) light exposure[31]. There are accumulating epidemiological, clinical, and basic data that support an immune-modulatory role for vitamin D in IBD[20]. On the other hand, IBD patients may be at a higher risk for vitamin D deficiency, thereby making this relationship a bidirectional one.

Bivariate analysis identified the following risk factors for vitamin D deficiency: (1) CD; (2) BMI > 30 kg/m^2^; (3) Age < 35 years; and (4) AA race. However, regression analysis showed that only AA race and BMI > 30 kg/m^2^ were significantly associated with vitamin D deficiency. While CD and vitamin D deficiency showed correlation, the relationship was not statistically significant likely due to insufficient numbers (*P* = 0.085). Similar findings have also been reported previously[15,32].

The prevalence of Obesity is increasing in the United States. According to the most recent obesity prevalence survey conducted by the Centers for Disease Control, greater than 35.1% United States adults and 35.5% of adults in Mississippi fall in the BMI > 35 kg/m^2^ category[33]. This is a potentially modifiable risk factor and may affect disease severity in IBD patients due to a pro-inflammatory effect[34] and through sequestration and/or volumetric dilution of vitamin D by adipose tissue[32,35]. Recently, Vimaleswaran et al[25] performed a bidirectional mendelian randomization analysis providing evidence for the role of obesity as a causal risk factor for the development of vitamin D deficiency.

Melanin in skin absorbs UVB light slowing the absorption and conversion of vitamin D3[36]. Therefore, African Americans are considered to be at increased risk for Vitamin D deficiency[37]. If vitamin D deficiency is a cause of IBD, then it could be theorized that African Americans would be at enhanced risk for IBD as well. Traditionally, African American risk for IBD is considered to be lower, not higher. This may in part be reflective of under-diagnosis within this population[26]. We found that race was a significant predictor of vitamin D concentrations based on the global test (*P <* 0.0001) with African American patients having a higher proportion of deficiency, while Whites and other races were four times less likely to have vitamin D deficiency.

Traditionally increasing age has been linked to vitamin D deficiency. This is related to multiple factors including: decreased metabolic activity of aging skin[23], reduced muscle mass that normally serves as a reservoir of vitamin D[30] and decreased sun exposure associated with residing in assisted living facilities[21].

Contrary to traditional belief, our initial analysis suggested that older age is protective against vitamin D deficiency (*P* = 0.0415). Future prospective studies are needed to help delineate the role of dietary, environmental and socio-economic factors that contribute to these findings.

While we feel that our study is well conducted and methodologically sound, we do recognize certain limitations. Our study is retrospective and data regarding all factors that affect vitamin D concentrations including: detailed dietary records, unreported supplement use, and cumulative sun exposure were not available for analysis. In many patients we struggled to find exact dates of symptom onset, history regarding smoking and alcohol use, objective assessment of symptoms including mayo clinical score or CDAI. Based on these issues we did not collect data regarding disease severity/need for surgery/complications/exact medication use etc. We do believe that the lack of this data does not undermine the validity of the presented data. This study includes patients from a single center and results may not be applicable to a different geographic area. Some of our findings may have achieved significance if we had studied a larger number of individuals. Despite these limitations, we are confident that this analysis accurately assesses the characteristics of vitamin D deficiency in a previously understudied population.

In summary, we hereby present data from a unique population in which disease state and diagnosis is significantly affected by dietary and socioeconomic status. Specifically, we show that BMI > 30 kg/m^2^ and AA race are associated with vitamin D deficiency in IBD and non-IBD patients. Future studies aimed at better understanding these differences may lead to improved disease outcomes.

## COMMENTS

### Background

The diagnosis and management of inflammatory bowel disease has made remarkable progress over the past decade. Both diagnostic as well as therapeutic paradigms are being constantly evaluated and improved. Despite the improved ability to identify and treat inflammatory bowel disease (IBD), it remains a significant source of morbidity and financial burden. The current treatment of IBD is also fraught with severe complications and adverse events. Therefore identification of preventable risk factors and development of risk-free novel agents is imperative to the progress of this field. In the recent past vitamin D has come to limelight for its immune modulating properties and has attracted the attention of IBD researchers worldwide, on one hand vitamin D deficiency is thought to play a role in pathogenesis of IBD and on the other hand it may be a consequence of IBD. There is significant disagreement amongst studies evaluating the relationship between IBD and vitamin D deficiency. This is at least partly due to confounding by factors that may affect both plasma vitamin D concentration and IBD. These factors may include age, race, gender, sex and body mass index (BMI). Additionally such data is quite sparse on African American patients. The population in Mississippi is unique in having a high percentage of African Americans as well as obese subjects. In this study we identified that a BMI > 30 and African American race are independent risk factors for vitamin D deficiency.

### Research frontiers

The association between vitamin D deficiency and pathogenesis of IBD is under investigation. Prior studies have significant disagreement. Better identification of confounding factors can help streamline future research and obtain concordant results. A more precise understanding of this relationship may lead to novel insights in to prevention and management.

### Innovations and breakthroughs

In this study the authors studied multiple possible risk factors for vitamin D deficiency in patients with and without IBD. The authors applied multiple regression to establish that BMI > 30 and African American race are associated with vitamin D deficiency in those with and without IBD. The authors also confirmed that age and gender did not significantly effect serum 25(OH)D concentrations in the studied population. This study confirms the findings of a large bi-directional Mendelian analysis. However literature is lacking in such data in African American patients and that makes our population unique.

### Applications

This study suggests that in future studies assessing the role of vitamin D deficiency in development of IBD as well as that of vitamin D in management of IBD should take into account the effects of race and BMI.

### Terminology

IBD or inflammatory bowel disease is a group of conditions of the small and large bowel characterized by chronic inflammation and a remitting and relapsing course. Crohn’s disease and ulcerative colitis are the two main forms of inflammatory bowel disease. While the mechanism of tissue destruction is autoimmune the exact etiology of these conditions is unknown. Over the past decade significant research has lead to better understanding and treatment options for these conditions however these treatments are associated with significant adverse events with average efficacy. The role of preventable risk factors such as vitamin D deficiency and obesity is currently under investigation.

### Peer-review

This is a well-written paper addressing the relevant scientific question; predictors of vitamin D deficiency in especially African American IBD patients. BMI > 30 and race and not diagnosis are found as predictors for vitamin D insufficiency, which is interesting and relevant to clinic practise.
